# Polymorphisms in *FFAR4* (*GPR120*) Gene Modulate Insulin Levels and Sensitivity after Fish Oil Supplementation

**DOI:** 10.3390/jpm7040015

**Published:** 2017-11-06

**Authors:** Bastien Vallée Marcotte, Hubert Cormier, Iwona Rudkowska, Simone Lemieux, Patrick Couture, Marie-Claude Vohl

**Affiliations:** 1 Institute of Nutrition and Functional Foods (INAF), Laval University, Quebec City, QC G1V 0A6, Canada; bastien.vallee-marcotte.1@ulaval.ca (B.V.M.); hubert.cormier.1@ulaval.ca (H.C.); simone.lemieux@fsaa.ulaval.ca (S.L.); patrick.couture@crchul.ulaval.ca (P.C.); 2 Endocrinology and Nephrology, CHU de Quebec Research Center, Quebec City, QC G1V 4G2, Canada; iwona.rudkowska@crchudequebec.ulaval.ca; 3 Department of Kinesiology, Laval University, Quebec City, QC G1V 0A6, Canada

**Keywords:** Gene–diet interactions, glycemic control, insulin resistance, omega-3 fatty acids, epigenetics, nutrigenenomics

## Abstract

The objective was to test whether *FFAR4* single nucleotide polymorphisms (SNPs) are associated with glycemic control-related traits in humans following fish oil supplementation. A total of 210 participants were given 3 g/day of omega-3 (n-3) fatty acids (FA) (1.9–2.2 g of eicosapentaenoic acid (EPA) and 1.1 g of docosahexaenoic acid (DHA)) during six weeks. Biochemical parameters were taken before and after the supplementation. Using the HapMap database and the tagger procedure in Haploview, 12 tagging SNPs in *FFAR4* were selected and then genotyped using TaqMan technology. Transcript expression levels were measured for 30 participants in peripheral mononuclear blood cells. DNA methylation levels were measured for 35 participants in leukocytes. In silico analyses were also performed. Four gene–diet interactions on fasting insulin levels and homeostatic model assessment of insulin resistance (HOMA-IR) index values were found. rs17108973 explained a significant proportion of the variance of insulin levels (3.0%) and HOMA-IR (2.03%) index values. Splice site prediction was different depending on the allele for rs11187527. rs17108973 and rs17484310 had different affinity for transcription factors depending on the allele. n-3 FAs effectively improve insulin-related traits for major allele homozygotes of four *FFAR4* SNPs as opposed to carriers of the minor alleles.

## 1. Introduction

Insulin resistance is a condition in which the response of sensitive tissues to insulin as well as glucose uptake are decreased [1]. Insulin resistance is a predictor of type 2 diabetes mellitus (T2DM), and individuals affected with T2DM have a higher cardiovascular risk [2]. Given that T2DM and cardiovascular diseases are major public health concerns worldwide, and especially in North America, where 40 million people are going to be affected by 2030, there is an urgent need to find new strategies to prevent the onset of T2DM, [3]. An omega-3 (n-3) fatty acid (FA) supplementation may exert beneficial effects on T2DM and cardiovascular risk, among others because n-3 FAs have the capacity to bind to the free fatty acid receptor 4 (FFAR4) [4].

*FFAR4*, commonly referred to G protein-coupled receptor 120 (*GPR120*), is a gene encoding for a protein that acts as a receptor for free fatty acids (FFAs) [5]. Unlike other FFARs, FFAR4 can only be activated by long chain FAs [6,7]. However, long-chain n-3 FAs, such as eicosapentaenoic acid (EPA) and docosahexaenoic acid (DHA), appear to be much more potent than α-linolenic acid (ALA) or arachidonic acid in stimulating FFAR4, and DHA is an even stronger FFAR4 agonist than EPA [6,7,8,9]. Numerous studies have shown that binding of n-3 FAs to FFAR4 is associated with various physiological activities having a stabilizing effect on metabolic homeostasis: (1) regulation of energy metabolism [10,11]; (2) anti-inflammatory effects [4]; (3) reduction of insulin resistance [12]; (4) facilitation of adipogenesis in adipose and liver tissues [13]; (5) maintenance of insulin sensitivity by inhibiting inflammation [12]; (6) pancreatic β-cells survival and proliferation [14]; and (7) stimulation of pancreatic insulin secretion (via the induction of glucagon-like peptide-1 (GLP-1) and cholecystokinin secretion from the intestine) [6,14,15]. *FFAR4* is also thought to be involved in the development of obesity in mice and humans [16].

Previous work from our laboratory has shown a large inter-individual variability in insulin-related traits such as the homeostatic model assessment of insulin sensitivity (HOMA-IS) index after a fish oil supplementation [17]. Accordingly, 99 individuals decreased their HOMA-IS (mean ± SD; −23.2 ± 14.3%) while 107 individuals increased their HOMA-IS (mean ± SD; 30.4 ± 48.4%) after the supplementation [17]. As the n-3 FA/FFAR4 complex plays an important role in insulin sensitivity and islet function, and *FFAR4* dysfunction was shown to be associated with insulin resistance, an obesity-related symptom of metabolic disorders, it becomes relevant to verify whether genetic variations within *FFAR4* contribute to the inter-individual variability observed in insulin-related traits [9,16,18,19].

The aim of the present study was to test whether *FFAR4* gene single nucleotide polymorphisms (SNPs) are associated with glycemic control-related traits in humans following n-3 FA supplementation. We hypothesize that carriers of minor alleles of *FFAR4* SNPs have altered glycemic control-related traits following fish oil supplementation.

## 2. Results

Allele frequencies of selected SNPs are presented in Table 1. All tagged SNPs were in Hardy–Weinberg equilibrium. Ninety-five percent of the genetic variability of *FFAR4* was covered (data not shown). Most selected SNPs were located in introns. One SNP, rs17108973, was located in the 3′ UTR region of *FFAR4* and another, rs17484310, was located in the 5′ UTR region. Subjects’ characteristics pre- and post-supplementation are presented in Table 2. The six-week n-3 FA supplementation increased fasting glucose levels (pre-suppl.: 4.95 ± 0.46; post-suppl.: 5.06 ± 0.49; *p* = 0.0004).

Using a stepwise bidirectional regression model adjusted for the effects of age, sex and body mass index (BMI), the contribution of *FFAR4* SNPs to the variance of baseline index values of homeostatic model assessment of insulin resistance (HOMA-IR) or insulin levels was estimated. Only rs17108973 explained a significant proportion of the variance of HOMA-IR (2.03%, *p* = 0.02) and insulin levels (3.0%, *p* = 0.005).

A large inter-individual variability between subjects was observed for post-supplementation fasting insulin levels. The contribution of *FFAR4* SNPs to this variability was assessed in a general linear model adjusted for the effects of age, sex, BMI and baseline fasting insulin levels. In this model, rs17108973 and rs11187537 were significantly associated with post-supplementation fasting insulin levels. β-estimates of rs17108973 and rs11187537 genotypes were respectively: CT/TT = 0.04 ± 0.02; CC = 0, *p* = 0.01; and CG/CC = 0.04 ± 0.02; GG = 0, *p* = 0.03.

**Figure 1:**
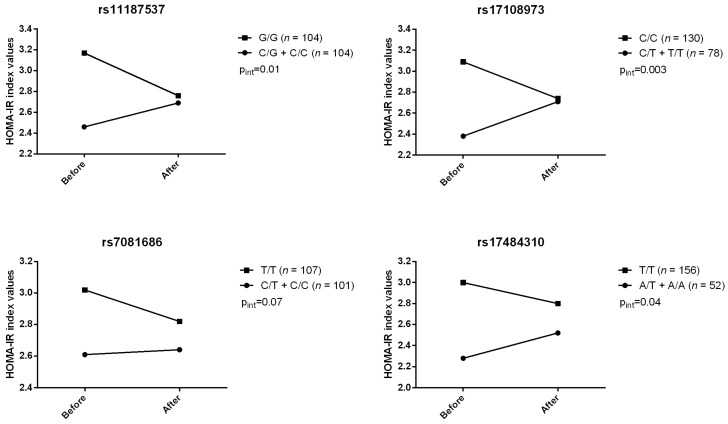
Gene–diet interactions on the homeostatic model of assessment of insulin resistance (HOMA-IR) index values. *p*-Values for differences in HOMA-IR changes between genotype groups: rs111887537, *p* = 0.015; rs17108973, *p* = 0.003; rs7081686, *p* = 0.0499; rs17484310, *p* = 0.038.

**Figure 2:**
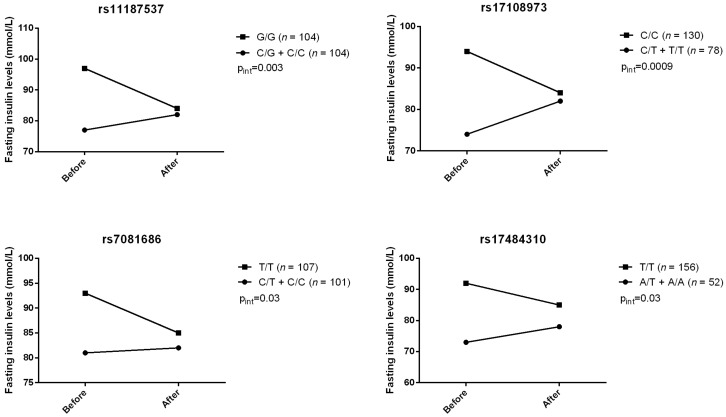
Gene–diet interactions on fasting insulin levels. *p*-Values for differences in insulin changes between genotype groups: rs111887537, *p* = 0.0045; rs17108973, *p* = 0.0012; rs7081686, *p* = 0.018; rs17484310, *p* = 0.031.

Gene–diet (supplementation) interaction effects were also tested using the MIXED procedure for repeated measures. Among 12 tagged SNPs, we observed four gene–diet interactions modulating HOMA-IR index (rs11187537, rs17108973, rs7081686, and rs17484310) (Figure 1). For these four SNPs, carriers of the minor allele had their HOMA-IR index increased after the n-3 FA supplementation. Four gene–diet interactions on fasting insulin levels after supplementation were also observed (Figure 2). Similarly, carriers of the minor allele had their insulin levels increased after the n-3 FA supplementation whereas homozygotes for the common genotype had a decrease. No gene–diet interaction with *FFAR4* SNPs on fasting glucose was observed. Pre- and post-supplementation fasting insulin levels and HOMA-IR index values according to genotype are presented in Supplementary Tables S1 and S2.

No effect of genotype on expression levels of *FFAR4* assessed on microarrays was observed. RNA splicing analyses identified two SNPs (rs11187527 and rs11187537) potentially located in splice sites. Splice site prediction was different depending on the allele for rs11187527, but not rs11187537, for which the acceptor site prediction score was similar with both alleles. No LD between studied SNPs neither other SNPs located in coding regions, nor SNPs previously identified in a genome-wide association study (GWAS) was observed. Transcription factor affinity predictions showed different affinity for several transcription factors depending on the allele of rs17484310 located in 5′UTR. The presence of its major allele led to an increased binding affinity to most transcription factors.

## 3. Discussion

In the present study, we tested whether SNPs within the *FFAR4* gene are associated with glycemic control-related traits in humans in response to an n-3 FA supplementation. We observed that carriers of the minor allele of several *FFAR4* SNPs increased their HOMA-IR index values and their fasting insulin levels after the six-week n-3 FA supplementation. Results also suggest that a single SNP, rs17108973, appeared to be driving gene–diet interactions on HOMA-IR and insulin levels. Moreover, rs17484310, which was also found to interact with the diet to exert effects on glycemic control-related traits, showed a different affinity to transcription factors depending on the allele. However, a transcription factor is unlikely to bind the region of this SNP due to its location (3’UTR). We also found that rs11187537 is positioned about 170 base pairs upstream a *FFAR4* exon and could potentially create an alternative splicing acceptor site. However, because alleles did not affect the splice site prediction score, the impact of rs11187537 on splicing may be modest. These outcomes suggest that the impact of *FFAR4* on the variability of the response of glycemic control-related traits to an n-3 FA supplementation may be predominantly attributable to the effect of rs17108973.

Numerous studies have shown that FFAs can bind to the G-protein coupled receptors such as GPR40, GPR41, GPR43, GPR84, and FFAR4 [20]. The association of n-3 FAs and FFAR4 results in an increase of the intracellular Ca^2+^ levels, the secretion of GLP-1and the activation of the extracellular signal-regulated kinase (ERK) family cascade [20,21]. The ERK cascade is a pathway that transfers extracellular signals mainly involved in the regulation of cell development, proliferation, differentiation, learning and sometimes apoptosis [22,23].

It has been shown that a particular SNP of *FFAR4*, rs116454156, is associated with obesity and insulin resistance in obese Europeans [10]. Bonnefond et al. observed a strong association between rs116454156 and increased fasting glucose levels [24]. In addition, rs116454156 contributed to decrease HOMA1-%B index values [24]. However, no association between this SNP and the risk of T2DM, variation of fasting insulin levels, HOMA-IR or oral glucose tolerance test (OGTT)-derived indices were reported in spite of the high statistical power [24]. The low allele frequency of rs116454156 (0.0150) might explain the absence of associations in their study [24]. This SNP has also been studied in obese children and adolescents and it was observed that carriers of the rare allele of rs116454156 had higher alanine transaminase (ALT) levels and higher ferritin levels, which is a marker of inflammation, than non-carriers [25]. rs116454156 is not in LD with any tagged SNP in the current study. Besides, it has been hypothesized that regulation of insulin secretion through FFAR4 activation does not result from direct stimulation of pancreatic β-cells, but rather from the inhibition of somatostatin release from δ-cells [26,27]. Stone et al. observed that the expression of *FFAR4* was predominant on mouse δ-cells and absent on β-cells [26,28]. The activation of FFAR4 down regulated glucose secretion via somatostatin production by islets [26,28]. Similar results were observed in a recent study by Yore et al., in which a FFAR4 agonist directly increased glucose-stimulated insulin secretion in human islets [29].

Studies with knockout mice reported mixed results. A recent study showed with *FFAR4* knockout mice that FFAR4 signaling was not required for n-3 FAs to exert their effects on insulin sensitivity and inflammation [30]. On the other hand, Oh et al. had obese mice fed with a high fat diet with or without n-3 FA supplementation and observed that n-3 FA did not inhibit inflammation and did not improve insulin sensitivity in *FFAR4* knockout mice as opposed to wild type mice [11]. These divergences may be attributable to diet composition. Indeed, differences between EPA and DHA could partly explain, besides genetic factors, the discrepancies observed in the effects of n-3 FAs on glucose metabolism. For instance, it has been observed in mice fed with a Western diet that EPA supplementation had a better preventive effect on glucose homeostasis deterioration and excessive accumulation of fat mass than ALA or DHA [31]. Besides, several other genes implicated in n-3 FA metabolism or glucose homeostasis may also affect the response of glycemic control-related traits to an n-3 FA supplementation.

According to McEwen et al., n-3 FAs are a judicious treatment option to improve the lipid profile in patients with T2DM [32]. In the present study, major allele homozygotes showed a more deteriorated profile, even at the baseline, but seem to be more responsive, thus showing a greater susceptibility to the effects of n-3 FAs as shown by decreased HOMA-IR index values after the six-week supplementation. Carriers of the minor allele showed lower HOMA-IR index values at the baseline. Thus, we can postulate that n-3 FAs are a good avenue of treatment for major allele homozygotes, but may not be as good for carriers of the minor allele.

### Strengths and Limitations

A key strength of the study was its experimental design where pharmacological doses of n-3 FAs were administered to participants. It would have been interesting to use an OGTT and to measure insulin secretion by dosing C-peptide. The current study does not allow investigating further the analyses of glycemic control-related parameters, suggesting that more studies are needed to better understand the impact of *FFAR4* SNPs on T2DM risk. Moreover, the intervention was not controlled by placebo and the design of did not allow comparing the differential effects of EPA and DHA on glycemic control-related traits. Differentiating EPA from DHA constitutes an important area for future research.

## 4. Materials and Methods

### 4.1. Subjects

A total of 254 unrelated subjects from the greater Quebec City metropolitan area were recruited through emails sent to University students and employees via advertisements in local newspapers. Inclusion criteria were as follows: (1) to be aged between 18 and 50 years; (2) being non-smoker; and (3) being free of pharmacologic lipid lowering treatment and/or metabolic disorders. Subjects who had taken n-3 FA supplements six months prior to the beginning of the study were excluded. A total of 210 subjects completed the supplementation protocol. Statistical power was previously calculated to observe a change in TG levels through n-3 FA supplementation. Individuals with CRP levels > 10 mg/L were excluded. The study was approved by the Université Laval and Centre hospitalier universitaire (CHU) de Québec ethics committees and was performed in accordance with the principles of the Declaration of Helsinki. All participants provided written and informed consent. This clinical trial was registered at clinicaltrials.gov (NCT01343342).

### 4.2. Study Design and Diets

To minimize the intra- and inter-variability in dietary intakes, a 2-week run-in period preceded the supplementation. During this run-in period, a registered dietitian gave individual dietary instructions in order to ensure that participants were in a stable condition before the beginning of the study. Participants also received nutritional recommendations according to the *Canada’s Food Guide* [33]. Participants were also asked to maintain their body weight stable throughout the whole research protocol. After the run-in period, each participant received n-3 FA capsules in sufficient quantity for the next six weeks. They were instructed to take five capsules/day of fish oil, providing a total of 3.0–3.3 g of n-3 FAs (1.9–2.2 g of EPA and 1.1 g of DHA). Before the run-in period, each participant completed a validated food-frequency questionnaire (FFQ) supervised by a registered dietitian [34]. This 91-item FFQ is based on typical food items found in North America. Moreover, they were asked to complete two 3-day food records - prior to and after the n-3 FA supplementation period. Dietary intakes data were analyzed using Nutrition Data system for Research software v.2011 (Nutrition Coordinating Center (NCC), University of Minnesota, Minneapolis, MN, USA).

### 4.3. Anthropometric Measurements

Body weight, height and waist circumference were measured at every visit in accordance with the Airlie Conference on the Standardization of anthropometric measurements [35]. BMI was calculated as weight per meter squared (kg/m^2^).

### 4.4. Biochemical Parameters

Blood samples were collected after a 12 h overnight fast and 48 h alcohol abstinence, from an antecubital vein into vacutainer tubes containing EDTA. Plasma total cholesterol and plasma TG concentrations were measured using enzymatic assays [36,37]. Infranatant (d > 1.006 g/mL) with heparin-manganese chloride was used to precipitate very low-density lipoprotein (VLDL) and low-density lipoprotein (LDL), and then to determine high-density lipoprotein (HDL)-cholesterol (HDL-C) concentrations [38]. Plasma C-reactive protein (CRP) was measured by nephelometry (Prospec equipment, Behring Diagnostic, Westwood, MA, USA) using a sensitive assay, as described previously [39]. Plasma concentrations of interleukin-6 (IL6) and tumor necrosis factor-α (TNF-α) were measured with high-sensitivity ELISA kits including: Human IL6 Quantikine HS ELISA Kit Minneapolis, MN, USA (R&D Systems, Minneapolis, MN, USA (HS600B)) and Human TNF-alpha Quantikine HS ELISA Kit (R&D Systems (HSTA00D)) [39]. Fasting insulin levels were measured using radioimmunoassay with polyethylene glycol separation [40]. Fasting glucose levels were enzymatically measured [41].

### 4.5. Genotyping of FFAR4 SNPs

**Figure 3:**
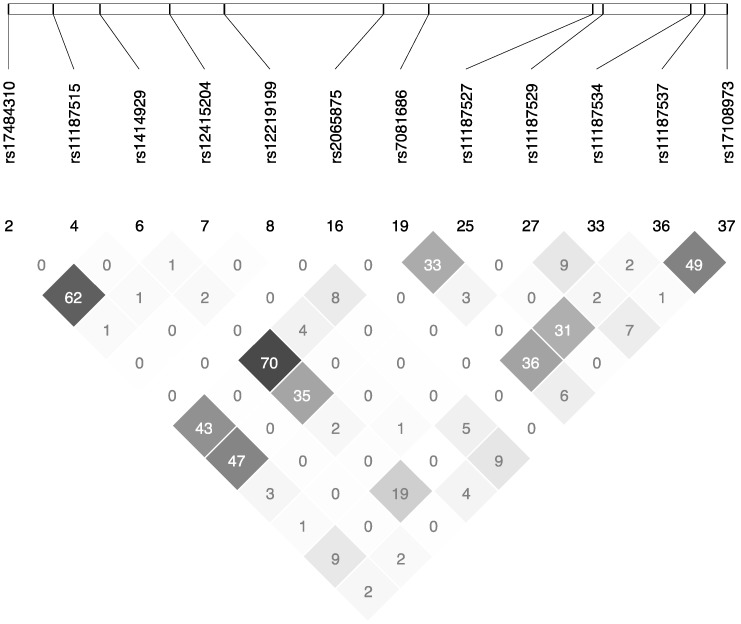
Linkage disequilibrium plot of selected SNPs in the *FFAR4* gene.

The International HapMap Project SNP database, based on the NCBI B36 assembly Data Rel 28. phase II + III, build 126, was used to identified *FFAR4* tagged SNPs [42]. We added 500-kilo base pairs downstream of *FFAR4* and 2500-kilo base pairs upstream to cover the 5’UTR and 3’UTR regions. Gene Tagger procedure in Haploview V4.2 was used to determine *FFAR4* tagged SNPs using a pairwise tagging (*r*^2^ ≥ 0.8) and a minor allele frequency ≥5%. Subsequently, we used the LD Plot procedure in Haploview V4.2 to examine the linkage disequilibrium out of the twelve *FFAR4* SNPs (Figure 3). Genomic DNA was prepared using the SIGMA GenElute Gel Extraction Kit (Sigma-Aldrich Co. St. Louis, MO, USA). *FFAR4* tagged SNPs (rs17484310, rs11187515, rs1414929, rs12415204, rs12219199, rs2065875, rs7081686, rs11187527, rs11187529, rs11187534, rs11187537, rs17108973) have been genotyped in 210 individuals using validated primers and TaqMan probes (Life Technologies Corporation, Burlington, ON, Canada) [43]. DNA was mixed with TaqMan Universal PCR Master Mix (Life Technologies Corporation), with a gene-specific primer and with probe mixture in a final volume of 10 μL (predeveloped TaqMan SNP Genotyping Assays). Genotypes were determined and analyzed using a 7500 RT-PCR System and ABI Prism SDS version 2.0.5 (Life Technologies Corporation).

### 4.6. Statistical Analyses

Statistical analyses were performed with SAS Genetics statistical software v9.3 (SAS, Toronto, ON, Canada). All genotype distributions were tested for any deviation from Hardy–Weinberg equilibrium using the ALLELE procedure. Significance testing for linkage disequilibrium coefficient D was obtained using a chi-square test, likelihood ratio and Fisher exact test (*p* ≤ 0.01). Since SNPs tested in complex diseases rarely account for a large proportion of the variance, results are thus presented without correction for multiple testing and using a *p* ≤ 0.05. Stepwise bidirectional regression model adjusted for the effects of age, sex, and BMI was conducted using the REG procedure (SAS v9.3) in order to test for associations between *FFAR4* SNPs and baseline index values of HOMA-IR or insulin levels. HOMA-IR was previously calculated according to the validated following equation: HOMA-IR = (FPI × FPG)/22.5, where FPI is fasting plasma insulin concentration (mU/L) and FPG is fasting plasma glucose (mmol/L) [44]. The GLM procedure adjusted for the effects of age, sex, BMI and baseline values of insulin levels was used to test for the effect of genotype on post-supplementation fasting insulin levels. The MIXED procedure for repeated measures adjusted for the effects of age, sex, and BMI was used to test for the effects of gene–diet interactions on glycemic control-related traits.

### 4.7. Transcriptomic and in Silico Analyses

The first 30 subjects who finished the intervention had their transcript expression measured in peripheral blood mononuclear cells using the Human-6 v3 Expression BeadChips (Illumina, San Diego, CA, USA). Transcriptomic analyses have been described in a previous paper [45]. The Berkeley Drosophilia Genome Project splice site prediction tool was used for RNA splicing analyses [46]. The SNP Annotation and Proxy Search (SNAP) online tool was used to evaluate LD between tagged and other non-tagged SNPs in the Northern Europeans from Utah (CEU) population [47]. The *r*^2^ was 80% and the distance limit was 500 kb. GWAS catalog was then used to verify if tagged and non-tagged SNPs have been previously identified in a GWAS [48]. Finally, the Transcription factor Affinity Prediction (TRAP) web tool for two sequences was used to evaluate transcription factor binding affinity differences between the alleles of tagged SNPs [49,50,51].

## 5. Conclusions

*FFAR4* variants, particularly rs17108973, can modulate the effect of n-3 FAs on insulin-related traits, particularly insulin resistance, after a six-week supplementation with high doses of n-3 FAs. The magnitude of changes varies substantially among individuals, meaning that what is good at the population level is not necessarily as good at the individual level. A better understanding of the phenomenon could allow the development of personalized dietary advice for the management of cardio-metabolic risk factors and diabetes.

## Figures and Tables

**Table 1:** Description of selected SNPs in *FFAR4.*

dbSNP No. ^1^	Sequence ^2^	Position	Alleles (Major/Minor)	AA	CC	CA	CG	CT	GA	GG	GT	AT	TT
				*n* ^3^ (%)									
rs1414929	GCA[A/T]GGA	Intron	A/T	127 (60.5)								69 (32.9)	14 (6.7)
rs12219199	ATG[C/T]GCC	Intron	C/T		176 (83.8)			31 (14.8)					3 (1.4)
rs2065875	TGC[C/T]CAC	Intron	C/T		194 (92.4)			16 (7.6)					
rs11187527	TTT[G/T]TAA	Intron	G/T							175 (83.3)	33 (15.7)		2 (1.0)
rs11187537	AGT[C/G]CAG	Intron	C/G		28 (13.4)		76 (36.4)			105 (50.2)			
rs17108973	AAC[C/T]TAT	UTR-3	C/T		131 (62.7)			62 (29.7)					16 (7.7)
rs7081686	TGA[C/T]GCT	Intron	C/T		19 (9.1)			84 (40.0)					107 (51.0)
rs17484310	AAA[A/T]CCG	UTR-5	A/T	6 (2.9)								47 (22.4)	157 (74.8)
rs11187529	GAG[C/T]GAT	Intron	C/T		194 (92.4)			16 (7.6)					
rs11187515	GAA[C/T]CAG	Intron	C/T		165 (78.6)			40 (19.1)					5 (2.4)
rs12415204	GGG[A/C]GAA	Intron	A/C	14 (6.7)	130 (61.2)	66 (31.4)							
rs11187534	CCC[A/G]AGC	Intron	A/G	2 (1.0)					42 (20.0)	166 (79.1)			

Allele frequencies from the FAS cohort, calculated with the ALLELE procedure in SAS Genetics v9.3; ^1^ SNP database (dbSNP) no. from HapMap Data Rel 28 Phase II + III, 10 August on NCBI b36 Assembly dbSNP b126 database; ^2^ Genes sequences from dbSNP short genetics variations NCBI reference assembly; ^3^ Number of subjects for each genotype.

**Table 2:** Characteristics of the study sample pre- and post-supplementation (*n* = 210).

Characteristics	Pre-Suppl.	Post-Suppl.	*p*-Value
Age, years	30.9 ± 8.7		-
Body mass index, kg/m^2 b,c^	27.8 ± 3.7	27.9 ± 3.8	0.005
Waist circumference, cm ^c^	93.3 ± 10.5	93.4 ± 10.7	0.53
Total cholesterol, mmol/L ^b,d^	4.75 ± 0.9	4.72 ± 0.94	0.42
HDL-cholesterol, mmol/L ^b,d^	1.44 ± 0.36	1.47 ± 0.40	0.004
LDL-cholesterol, mmol/L ^d^	2.76 ± 0.81	2.78 ± 0.85	0.47
TG, mmol/L ^b,d^	1.21 ± 0.63	1.02 ± 0.52	<0.0001
Apolipoprotein B, g/L ^d^	0.84 ± 0.24	0.87 ± 0.24	0.003
Fasting insulin, ρmol/mL ^b,d^	87.2 ± 75.7	83.6 ± 40.8	0.81
Fasting glucose, mmol/L ^d^	4.95 ± 0.46	5.05 ± 0.49	0.0004
CRP, mg/L ^b,d^	2.59 ± 3.92	2.66 ± 4.32	0.93
TNF-α, ρg/mL ^b,d^	1.68 ± 1.42	1.68 ± 1.26	0.96
IL-6, ρg/mL ^b,d^	1.38 ± 1.13	1.34 ± 0.99	0.63

Values are means ± SD; ^a^ *p*-values derived from a repeated MIXED procedure; ^b^ Values are log_10_-transformed; ^c^ Values adjusted for age and sex; ^d^ Values adjusted for age, sex, and body mass index. HDL: high-density lipoprotein; LDL: low-density lipoprotein; TG: triglyceride; CRP: C-reactive protein; TNF-α: tumor necrosis factor-α; IL-6: interleukin-6.

## Supplementary Materials

The following are available online at http://www.mdpi.com/2075-4426/7/4/15/s1, Table S1: Pre- and post-supplementation fasting insulin levels according to genotype for tagging SNPs in *FFAR4* (*n* = 208); Table S2: Pre- and post-supplementation HOMA-IR index values according to genotype for tagging SNPs in *FFAR4* (*n* = 208).
