# Supplementary material for: Polymorphisms in FFAR4 (GPR120) Gene Modulate Insulin Levels and Sensitivity after Fish Oil Supplementation
Source: J Pers Med. 2017 Nov 6;7(4):15. doi: 10.3390/jpm7040015 (PMC5748627; doi:10.3390/jpm7040015)
Supplement: Supplementary file 1 [file jpm-07-00015-s001.pdf]

Supplementary Table 1. Pre- and post-supplementation fasting insulin levels according to genotype for tagging SNPs in *FFAR4* (n = 208).

	Pre-Supplementation		Post-Supplementation		
**SNP**	**Homozygotes**	**CMA**	**Homozygotes**	**CMA**	***p*-value (Gene-Diet)**
rs11187537	97.07 ± 10.05	77.22 ± 2.78	84.63 ± 4.82	82.63 ± 3.02	0.003
rs17108973	94.74 ± 8.16	74.48 ± 2.84	84.32 ± 4.02	82.47 ± 3.53	0.0009
rs7081686	93.14 ± 8.65	80.80 ± 5.71	85.36 ± 4.22	81.80 ± 3.77	0.03
rs17484310	91.88 ± 6.83	72.94 ± 4.01	85.42 ± 3.57	78.26 ± 3.66	0.03
rs1414929	89.55 ± 7.33	83.38 ± 7.07	84.75 ± 3.72	81.87 ± 4.37	0.2
rs12219199	88.18 ± 6.05	81.66 ± 8.11	82.91 ± 2.88	87.36 ± 9.30	0.2
rs2065875	86.80 ± 5.54	91.60 ± 15.15	83.07 ± 2.76	90.73 ± 17.14	1.0
rs11187527	88.93 ± 6.17	78.00 ± 5.49	83.88 ± 3.22	82.32 ± 5.40	0.3
rs11187529	85.83 ± 5.15	102.93 ± 29.43	83.76 ± 3.01	81.93 ± 7.21	0.6
rs11187515	84.62 ± 3.98	96.31 ± 19.61	84.88 ± 3.15	79.08 ± 6.43	0.3
rs12415204	88.37 ± 7.96	85.13 ± 4.74	84.78 ± 3.74	81.74 ± 4.32	0.2
rs11187534	82.67 ± 3.82	103.81 ± 20.29	81.92 ± 2.52	89.95 ± 9.55	0.4

Values are mean ± SE; CMA: Carriers of the minor allele; *p*-values derived from a repeated MIXED procedure adjusted for age, sex and BMI.

Supplementary Table 2. Pre- and post-supplementation HOMA-IR index values according to genotype for tagging SNPs in *FFAR4* (n = 208).

	Pre-Supplementation		Post-Supplementation		
**SNP**	**Homozygotes**	**CMA**	**Homozygotes**	**CMA**	***p*-value (Gene-Diet)**
rs11187537	3.17 ± 0.39	2.46 ± 0.09	2.76 ± 0.16	2.69 ± 0.10	0.01
rs17108973	3.08 ± 0.31	2.37 ± 0.09	2.74 ± 0.13	2.71 ± 0.12	0.003
rs7081686	3.02 ± 0.33	2.60 ± 0.22	2.81 ± 0.15	2.64 ± 0.12	0.07
rs17484310	3.00 ± 0.26	2.28 ± 0.13	2.80 ± 0.12	2.51 ± 0.12	0.04
rs1414929	2.89 ± 7.33	2.715 ± 0.27	2.77 ± 3.72	2.66 ± 0.14	0.2
rs12219199	2.85 ± 0.23	2.63 ± 0.25	2.71 ± 0.10	2.83 ± 0.28	0.4
rs2065875	2.80 ± 0.21	3.01 ± 0.48	2.71 ± 0.10	2.98 ± 0.52	0.9
rs11187527	2.89 ± 0.23	2.44 ± 0.18	2.74 ± 0.11	2.64 ± 0.18	0.3
rs11187529	2.75 ± 0.19	3.57 ± 1.24	2.73 ± 0.10	2.63 ± 0.27	0.4
rs11187515	2.71 ± 0.15	3.19 ± 0.76	2.77 ± 0.10	2.57 ± 0.23	0.3
rs12415204	2.89 ± 0.31	2.69 ± 0.14	2.76 ± 0.12	2.67 ± 0.15	0.2
rs11187534	2.66 ± 0.14	3.40 ± 0.78	2.67 ± 0.08	2.93 ± 0.32	0.5

Values are mean ± SE; CMA: Carriers of the minor allele; *p*-values derived from a repeated MIXED procedure adjusted for age, sex and BMI.
